#  Effects of Phenylglyoxal and N-ethylmaleimide Concentration on Mycophenolic Acid Production by *Penicillium brevi-compactum *ATCC16024

**Published:** 2016

**Authors:** Fatemeh Ardestani

**Affiliations:** *Department of Chemical Engineering, Qaemshahr Branch, Islamic Azad University, Qaemshahr, Iran.*

**Keywords:** Antibiotic, Aerobic fermentation, Immunosuppressive, Mycophenolic acid, *Penicillium brevi-compactum*

## Abstract

Mycophenolic acid is a secondary extracellular metabolite of *Penicillium* strains with numerous pharmaceutical properties such as antibiotic and immunosuppressive uses. The aim of this work is the survey of the effect of phenylglyoxal and n-ethylmaleimide concentration in culture medium on mycophenolic acid production by *Penicillium brevi-compactum* ATCC16024 was investigated. Batch submerged fermentation was performed in 250 mL shake flasks at 24 °C and 200 rpm in a rotary shaker for 300 h using a basic culture medium containing different concentrations of phenylglyoxal and n-ethylmaleimide ranged from 0 to 20 mg. L^-1^. For the basic medium without any amounts of phenylglyoxal and n-ethylmaleimide (control), maximum MPA production, product yield and productivity of process was in order, 1.5042 g. L^-1^, 20.3 mg. g^-1^ consumed glucose and 5.37 mg. L^-1^h^-1^. Maximum produced MPA of 2.9032 g. L^-1^, MPA yield of 39.23 mg. g^-1^ of consumed glucose, productivity of 10.37 mg. L^-1^ h^-1^ and total enhancement of 93.11% was obtained when the culture medium was contained 18 mg. L^-1^ of phenylglyoxal, represented more than 93% raising in compare to control. Maximum MPA concentration, yield and productivity in order was obtained 3.1123 g. L^-1^, 42.06 mg. g^-1^ of consumed glucose and 11.11 mg. L^-1^ h^-1^, with using 6 mg. L^-1^ of n-ethylmaleimide. N-ethylmaleimide was caused to 2.138 folds (106.89%) increase in MPA production by *P. brevi-compactum* ATCC16024.

## Introduction

Mycophenolic acid ((4E)-6-(4-Hydroxy-6-methoxy-7-methyl-3-oxo-1,3-dihydro-2-benzofuran-5-yl)-4-methylhex-4-enoic acid) and its derivatives such as mycophenolate mofetil are new drugs with numerous pharmaceutical properties such as immunosuppressive and decrease the incidence of graft rejection after organ transplantation (1), anti-inflammatory (2), anti-virus (3), anti-tumor (4, 5), debarment of chronic allograft failure and effective for the various inflammatory diseases such as glomerulopathies, systemic lupus erythematosus and systemic vasculitis (6). MPA is produced by different species of *Penicillium* and also by some other microbial strains such as *Byssochlamys nivea* in submerged and solid state fermentation processes (7, 8, 9). Different operational modes such as free and immobilized cells in submerged cultures (10), packed bed bioreactors (11) and fed-batch systems (12) were used for MPA production. Due to the totally low production yields in different conducted MPA production process, finding any improver method or material could be very valuable. 

Inhibitory properties of phenylglyoxal (oxo (phenyl) acetaldehyde 1-phenylethanedione, C_8_H_6_O_2_) on transketolase and phosphoenolpyruvate carboxykinase, two key enzymes in MPA biosynthesis pathway (13, 14, 15); as well as inactivation of four other key enzymes of the mentioned pathway: squalene synthase, NADH dehydrogenase, pyrroline-5-carboxylate reductase and hydroxymethylglutaryl-CoA synthase by N-ethylmaleimide (C_6_H_7_NO_2_) created a primary idea to investigate the effects of these compounds on MPA biosynthesis in* Penicillium brevi-compactum *(16). 

In this study, the effects of phenylglyoxal and n-ethylmaleimide concentration on MPA production by *Penicillium brevi-compactum* ATCC16024 were evaluated. 

## Material and Methods

*Material*

Phenylglyoxal hydrate 97% and n-ethylmaleimide crystalline 98% were prepared from Sigma-Aldrich Co. LLT. Analytical standard mycophenolic acid (C_17_H_20_O_6_) powder with more than 98% purity was prepared from Sigma-Aldrich Co. LLT. All other chemicals and microbial cultures were prepared from Merck and Sigma-Aldrich. 

*Microorganism and inoculum preparation*

*Penicillium brevi-compactum* ATCC16024 was obtained from American Type Culture Collection (ATCC) as an ampoule containing freeze-dried viable cells suspended in cryoprotectant. The stock culture was maintained on the Potato dextrose agar (PDA) slants at 4 °C. PDA containing petri dishes were used for preparation of working cultures. After inoculation, petri dishes were incubated at 24 °C for 3 days. The cell suspension was made by collection of spores grown on petri plates by shaving and extracting the spores with sterile water (8). 

The number of spores in suspension was counted by Thoma lam and adjusted to 10^7^- 10^8^ spores per mL. Spore suspension was used as the inoculums for shake flask. For fermentation process, 1 mL of spore suspension (~10^8^ per mL) was inoculated to 50 mL culture medium contained in 250 mL shake flasks (8). 

*Medium composition*

The basic medium composition was included (g. L^-1^): glucose, 80; glycine, 9; enzymatically hydrolyzed casein, 15; methionine, 0.5; KH_2_PO_4_, 5; MgSO_4_.7H_2_O, 1; and 1 ml. L^-1^ trace element mixture including (g. L^-1^): FeSO_4_·7H_2_O, 2.2; CuSO_4_·5H_2_O, 0.3; ZnSO_4_·7H_2_O, 2.4; MnSO_4_·4H_2_O, 0.16; and KMoO_4_, 0.2 (8). 

3 shake flasks for control (basic medium without any amounts of phenylglyoxal and n-ethylmaleimide, 36 shake flasks containing basic medium with different phenylglyoxal concentrations (0.5 to 20 mg. L^-1^, each treatment in three repeat) and also 36 shake flasks containing basic medium with different n-ethylmaleimide concentrations (0.5 to 20 mg. L^-1^, each treatment in three repeat) were prepared. All shake flasks were autoclaved at 121 °C for 15 min. Amino acids (here glycine and methionine) are heat sensitive compounds and may be decompose in the presence of other medium components at high temperatures. Thus they strolled separately by microfiltration (0.2 μm, Millipore, USA) and then added to the shake flasks contents under sterile conditions. All culture medium pH was adjusted on 6 using 2 N HCl or NaOH solutions (8). 

**Figure 1:**
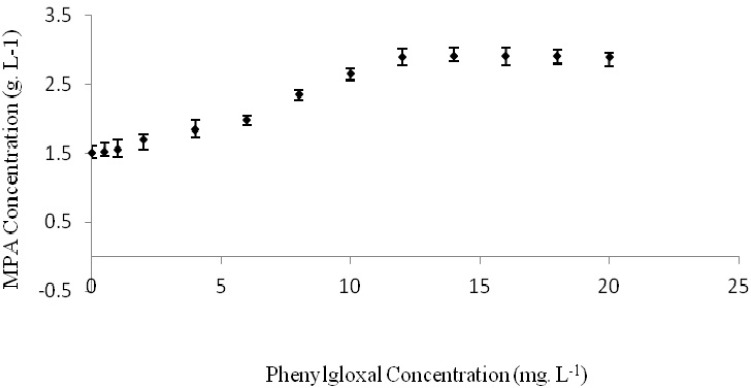
Effects of phenylglyoxal on MPA production by *Penicillium* *brevi-compactum* ATCC16024 at 24 °C and pH=6 with 200 rpm agitation speed in a rotary shaker for 300 h incubation time

**Figure 2:**
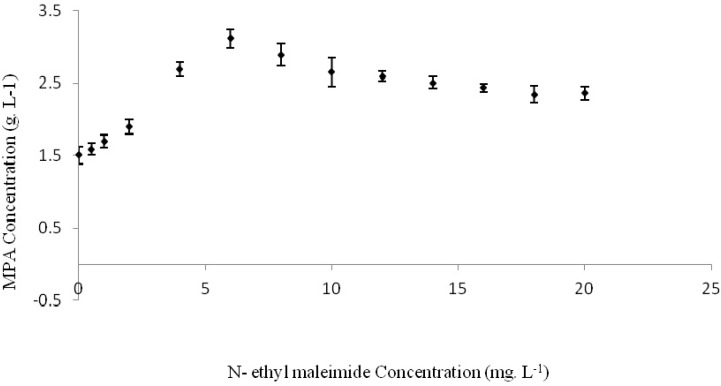
Effects of n-ethylmaleimide on MPA production by *Penicillium* *brevi-compactum* ATCC16024 at 24 °C and pH=6 with 200 rpm agitation speed in a rotary shaker for 300 h incubation time

*Fermentation process*

Batch fermentation was performed in a rotary shaker incubator (JAHL- JSH 20LUR, IRAN). Inoculated shake flasks were incubated at 24 °C with 200 rpm agitation speed in a rotary shaker for 300 h. MPA production was investigated at the end of cultivation time. The effects of phenylglyoxal and n-ethylmaleimide concentration on MPA production were evaluated separately as explained in medium composition section. MPA concentration in each shake flask was measured after 280 h. MPA was extracted from culture medium using a liquid adsorption chromatography column (GIS column packed with high purity silica gel, Shimadzu, Japan). For this purpose, the first, medium pH was adjusted on 7 and cell biomass separated by centrifugation at 10000 rpm for 10 min. Then, adsorption chromatography was conducted and at the next step, the column was eluted by methanol. MPA white powder was purified from eluted solution by precipitation using chilled water at pH = 2 and drying in oven at 65 °C until constant weight (17). 

*Analytical methods*

All contents of each flask were used as sample for MPA concentration measurement. At the end of incubation time (after 280 h), MPA concentration was measured for filtered samples through a 0.2 μM filter (Millipore, USA). High performance liquid chromatography method (HPLC, Shimadzu, Japan) with a C_18_ column at 40 °C was used to determine MPA concentration (18). 0.5 mL. min^-1^ of a mixture in equal proportion of 0.1 M potassium hydrogen phosphate and acetonitrile at pH = 3.0 was used as the mobile phase. The applied wavelength of UV detector was 250 nm. Sample injection volume was 50 µL (19). HPLC grade MPA was used as standard material to determine MPA concentration. Methanol was used as the main solvent to prepare the stock (1 mg. mL^-1^) and working (2.5–250 µg. mL^-1^) standards of MPA. All measurements were repeated for three times and the mean values are considered as the experimental results (8). 

Colorimetric method by using spectrophotometer (Unico 2100, USA) at 540 nm was used to determine glucose concentration for each shake flask contents at the end of incubation time (8). 

*Statistical data analysis*

SPSS 22 software was used for data analysis. Two ways analysis of variance (ANOVA) and Fishers Least Significant Difference (LSD) test were applied to compare the mean values of the produced MPA concentration between all treatments. 

P≤0.05 was considered as significant difference between each treatment with other experiments. 

## Results and Discussion

*P. brevi-compactum* ATCC16024 was appeared as pellets after about 24 h from incubation. These pellets served during the fermentation process until 250 h (the end of the stationary phase) and then broke and disappeared. The major amount of glucose was consumed in the early 180 h of incubation, tropho-phase stage of *P. brevi-compactum* growth. Then, at the idio-phase stage of growth, glucose consumption rate was decreased and reached to an approximate constant value. Glucose concentration was decreased from 10.51 g. L^-1^ at the start of the stationary phase to 4.27 g. L^-1^ at the end of this phase. MPA production as a secondary metabolite was occurred between 180 to 280 h of incubation time. 

*Effect of phenylglyoxal on MPA production*

Maximum produced MPA in the basic medium without any amounts of phenylglyoxal, n-ethylmaleimide and 8-hydroxycoinoline-5-sulfonic acid was reached to 1.5042 g. L^-1^, at 280 h after incubation. MPA production yield was calculated 20.3 mg. g^-1^ of consumed glucose and its productivity was determined as 5.37 mg. L^-1^ h^-1^. 

Some separate experiments were conducted using basic medium enriched by different concentrations of phenylglyoxal. MPA concentration was determined after 280 hours for each experiment (Figure 1.). Maximum produced MPA of 2.9032 g. L^-1^, was obtained when the culture medium was contained 18 mg. L^-1^ of phenylglyoxal. In this condition, MPA yield and productivity were 39.23 mg. g^-1^ of consumed glucose and 10.37 mg. L^-1^ h^-1^, respectively. These values were higher than MPA production in basic medium without any phenylglyoxal as 93.11% (**Table 1**.). However, statistical analysis of results didn’t show any considerable differences between treatments with 12 to 20 mg L^-1^ of phenylglyoxal (p>0.05). 

Thus, phenylglyoxal was caused to two times increase in MPA production by *P. brevi-compactum* ATCC16024 under the applied conditions. This impact may be related to phenylglyoxal effect on inhibition of certain enzymes involved in the MPA biosynthetic pathway of *P. brevi-compactum*. Based on some previous reports, phenylglyoxal has an inhibitory effect on transketolase (13, 14), the enzyme responsible for the conversion of xylulose-5-phosphate (X5P) and ribose-5-phosphate (R5P) to sedoheptulose-7-phosphate (S7P) and glyceraldehydes-3- phosphate (GAP). This is a key biochemical reaction in MPA biosynthesis pathway. Checkrein this reaction can probably lead to a raise in the main reactions responsible to final MPA biosynthesis in *P. brevi-compactum* biochemical pathway. Phenylglyoxal also has an inhibitory effect on phosphoenolpyruvate carboxykinase, the enzyme responsible for the conversion of oxaloacetic acid (OAA) to phosphoenolpyruvate (PEP) (15). OAA is a key metabolite covert to acetate, methionine and mevalonate. Mycophenolic acid molecule is made from acetate-derived compounds, methionine and mevalonic acid (20, 21). Then, the most OAA aggregation leads to higher production of the aforesaid components. Based on some previous reports, adding methionine and acetate to *P. brevi-compactum* culture medium could cause to an improved MPA production yield and process productivity (22, 23). The same reports were presented even for some other microbial species such as *Acremonium chrysogenum* and other antibiotic production process (24).

*Effect of n-ethylmaleimide on MPA production*

Effects of different concentrations of n-ethylmaleimide on MPA production by *Penicillium brevi-compactum *was investigated as separate experiments and produced MPA concentration was assayed 280 h after incubation (Figure 2.). Maximum produced MPA of 3.1123 g. L^-1^, was obtained when the culture medium was contained 6 mg. L^-1^ of n-ethylmaleimide. Thus, maximum MPA yield and productivity was in order 42.06 mg. g^-1^ of consumed glucose and 11.11 mg. L^-1^ h^-1^. These values were higher than MPA production in basic medium without any n-ethylmaleimide as 106.9% (Table 2). Adding n-ethylmaleimide more than 6 mg. L^-1^ not only had not more positive effects on MPA biosynthesis by *Penicillium brevi-compactum*, but also showed a negative impact. Statistical analysis of results showed considerable difference between the treatments containing of 6 mg. L^-1 ^n-ethylmaleimide and other studied treatments. 

Thus, n-ethylmaleimide was caused to 2.138 folds increase in MPA production by *P. brevi-compactum* ATCC16024 in the performed fermentation process. N-ethylmaleimide has inhibitory effect on four key enzymes involved in MPA biosynthesis biochemical pathway: squalene synthase, NADH dehydrogenase, pyrroline-5-carboxylate reductase and hydroxymethylglutaryl-CoA synthase. Squalene synthase is the main enzyme responsible for ergosterol (ERG) biosynthesis from farnesyl-diphosphate (F-PP) and s-adenosyl methionine (SAM) as presented in Equation 1that inhibit by n-ethylmaleimide (16). Thus, much amounts of methionine will consume in MPA biosynthesis reactions. Also, F-PP convert to 6-farnesyl-5, 7-dihydroxy-4-methylphethalide, the main precursor of MPA. On the other word, this inhibition may cause to an increase in MPA production (25). 

1F-PP+ + 6NADPH+ 6O_2_+ 2H+ 1SAM →1ERG+ 2CO_2_+ 6NADP+ 3H_2_O+ 1SA-HCYS          (Eq. 1)

The second inhibited enzyme is NADH dehydrogenase, the biocatalyst undertaking to run the Equation 2. in the section of oxygen transfer chain. N-ethylmaleimide blocks the phosphate porter by reacting with SH groups and prevents respiration by coupled mitochondria and phosphate-mediated swelling. Its inhibitory effect on NADH dehydrogenase was reported by some previous researches (26) probably could promote MPA biosynthesis pathway (27, 28). 

1NAD(P)H+ 1O_2_+ 2(P/O) ADP+ 2(P/O) P_i_→ 2(P/O) ATP+ 1NAD(p)          (Eq. 2) 

Pyrroline-5-carboxylate reductase catalyzes the conversion of glutamine (GLU) to proline (PRO) as presented in Equation 3. Less activity of pyrroline-5-carboxylate reductase affected by n-ethylmaleimide (29), cause to more conversion rate of glutamine to methionine, one of the main precursors of MPA and certainly, final result can be more MPA biosynthesis in the cell. 

1GLU+ 1ATP+ 2NADPH+ 2H→ 1PRO+ 1H_2_O+ 2NADP+ 1ADP+ 1P_i_          (Eq. 3)

N-ethylmaleimide showed negative effects on the activity of hydroxymethylglutaryl-CoA synthase, the main enzyme to operate the conversion of Acetoacetyl-COA (AACOA) to 3-hydroxy-3-methylglutaryl COA (3H3MGCoA) as presents in Equation 4. Inhibition of this reaction, unlike previous cases, leads to a decrease in MPA biosynthesis. 3H3MGCoA is a precursor for mevalonate synthesis, one of the main parts of MPA molecule. This may be the reason of lower MPA production at higher contents of n-ethylmaleimide.

1ACCOA+ 1H_2_O+ 1AACCOA→ 1 3H3MGCoA+ 1CoA+ 1CO_2_           (Eq. 4) 

## Conclusion

MPA production by *Penicillium brevi-compactum* ATCC16024 with different content of phenylglyoxal and n-ethylmaleimide concentration was evaluated in batch submerged fermentation. Both two applied agents showed positive impacts on MPA biosynthesis. MPA production was enhanced for 93.11% with adding 18 mg. L^-1^ of phenylglyoxal to the produced cell culture. While, adding only 6 mg. L^-1^ of n-ethylmaleimide caused to 106.9% raise in MPA production. 
